# Oncogenic roles of the SETDB2 histone methyltransferase in gastric cancer

**DOI:** 10.18632/oncotarget.11625

**Published:** 2016-08-26

**Authors:** Taketo Nishikawaji, Yoshimitsu Akiyama, Shu Shimada, Kazuyuki Kojima, Tatsuyuki Kawano, Yoshinobu Eishi, Yasuhito Yuasa, Shinji Tanaka

**Affiliations:** ^1^ Department of Molecular Oncology, Graduate School of Medical and Dental Sciences, Tokyo Medical and Dental University, Tokyo, Japan; ^2^ Department of Surgical Oncology, Graduate School of Medical and Dental Sciences, Tokyo Medical and Dental University, Tokyo, Japan; ^3^ Department of Surgery, Graduate School of Medical and Dental Sciences, Tokyo Medical and Dental University, Tokyo, Japan; ^4^ Department of Human Pathology, Graduate School of Medical and Dental Sciences, Tokyo Medical and Dental University, Tokyo, Japan

**Keywords:** gastric cancer, histone methyltransferase, H3K9me3, SETDB2

## Abstract

SETDB2 is a histone H3 lysine 9 (H3K9) tri-methyltransferase that is involved in transcriptional gene silencing. Since it is still unknown whether SETDB2 is linked to carcinogenesis, we studied alterations and functions of SETDB2 in human gastric cancers (GCs). SETDB2 protein was highly expressed in 30 of 72 (41.7%) primary GC tissues compared with their normal counterparts by immunohistochemistry. SETDB2 overexpression was significantly associated with the late stage of GCs (*P*<0.05) and poor prognosis of GC patients (*P*<0.05). The GC cell lines with *SETDB2* knockdown and overexpression significantly decreased and increased cell proliferation, migration and invasion, respectively (*P*<0.05). Knockdown of *SETDB2* in MKN74 and MKN45 cells reduced global H3K9 tri-methylation (me3) levels. Microarray analysis indicated that expression of *WWOX* and *CADM1,* tumor suppressor genes, was significantly enhanced in MKN74 cells after *SETDB2* knockdown. Chromatin immunoprecipitation assays showed that the H3K9me3 levels at the promoter regions of these two genes corresponded to the SETDB2 expression levels in GC cells. Moreover, ectopic SETDB2 protein was recruited to their promoter regions. Our data suggest that SETDB2 is associated with transcriptional repression of *WWOX* and *CADM1*, and hence overexpression of SETDB2 may contribute to GC progression.

## INTRODUCTION

Gastric cancer (GC) is the third leading cause of death from cancer in the world, and the incidence is still high [1]. Genetic and epigenetic alterations are involved in gastric carcinogenesis; for example, mutations of the *CDH1* and *p53* tumor suppressor genes (TSGs), and hypermethylation at the CpG island promoter region of TSGs have been detected in GCs [2, 3].

Tri-methylation mark of histone H3 lysine 4 (H3K4me3) is involved in transcriptional activation of genes, whereas tri-methylation marks of H3K9 (H3K9me3) and K27 (H3K27me3) are associated with gene silencing in the transcriptional process. These histone methylations are catalyzed by histone methyltranseferases (HMTs) containing an evolutionally served SET (SuVar3-9, enhancer of Zeste, Trithorax) domain [4]. Among them, *SETDB1, SUV39H1* and *EZH2* are reported to be frequently over-expressed in various cancers, such as GCs, those of which functions are shown as oncogenic activities [5–8]. We have recently reported that expression of SET7/9, a histone H3K4 mono-methyltransferase, was reduced in advanced GCs [9]. Thus, expression changes of histone modification genes are known to play an important role in tumor development.

SETDB2 have been characterized as H3K9 tri-methyltrasferases that contain a bifurcated SET domain, a pre-SET domain and a Methyl-CpG binding domain [10, 11]. Setdb2 restricts dorsal organizer territory and regulates structural left-right asymmetry of visceral organs and nervous system by suppressing the activity of *fibroblast growth factor 8* in zebrafish embryo [10]. Moreover, it was reported that knockdown of Setdb2 in zebrafish embryo induced abnormal convergence and extension movements during gastrulation, resulting in anterior-posterior shortening [12], indicating an essential role in embryonic development. Although SETDB2 is known to be associated with chromosomal condensation and segregation through H3K9 tri-methylation (me3) regulation during mitosis [11], the molecular mechanism by which SETDB2 mediates transcriptional gene silencing remains unclear. Furthermore, there has not been reported the evidence presented of SETDB2 alterations in cancers. In this study, we aimed to clarify whether SETDB2 is linked to carcinogenesis. Since we found that SETDB2 was often overexpressed in both primary GC tissues and GC cell lines, we further analyzed its biological functions in GC cells.

## RESULTS

### Overexpression of SETDB2 in primary GC tissues

**Figure 1:**
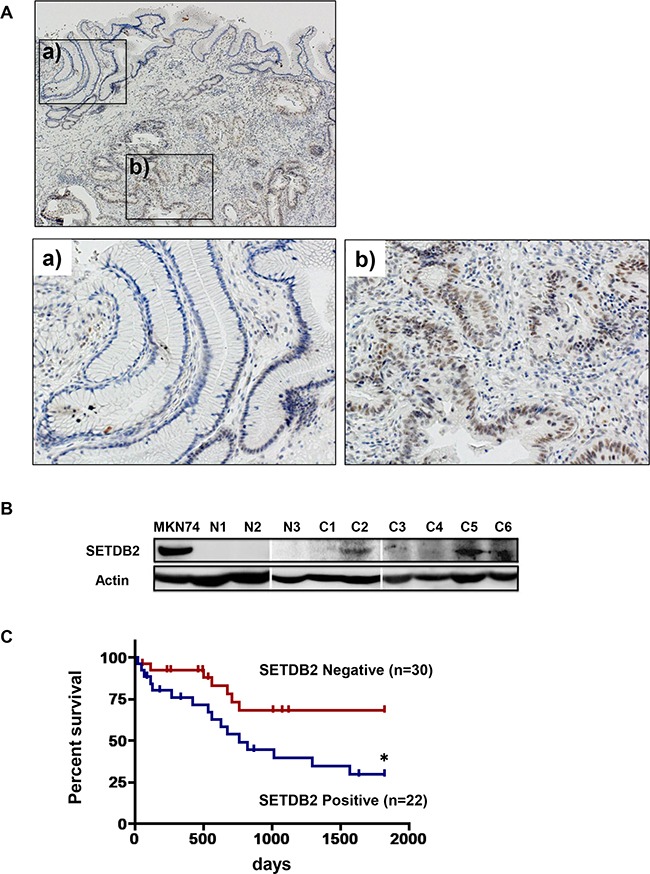
Analyses of SETDB2 protein expression in primary GC tissues **A.** Immunohistochemical staining of SETDB2 protein expression. The staining is representative of 72 primary GC tissues. The images at bottom are magnifications of the boxed regions of non-cancerous (a) and tumor (b) tissues in the top image. **B.** Western blot analysis of the expression levels of SETDB2 protein in primary GC tissues and non-cancerous stomach tissues. Representative data are shown. Actin expression was analyzed as an internal control. Expression in the MKN74 cell lines was used as a positive control (Figure 2A). N; non-cancerous stomach tissue, C; human gastric cancer. **C.** Kaplan Meier analysis of overall survival of gastric cancer patients according to SETDB2 positive or negative expression in the tumor tissue (*: *P*<0.05).

We analyzed the expression of SETDB2 protein in a set of 72 primary GC tissues by immunohistochemical staining (IHC). SETDB2 expression was not detected in any non-cancerous stomach tissues, whereas SETDB2 was strongly expressed in the nuclei of 30 GC tissues (41.7%, Figure 1A). Western blot analysis showed that SETDB2 protein was expressed in four of 12 primary GC tissues (33.3 %) but not in any of the non-cancerous stomach tissues tested (Figure 1B). To evaluate the significance of SETDB2 alteration in primary GCs, we studied the relationship between SETDB2 overexpression and clinicopathological factors of GCs. SETDB2 was significantly overexpressed in advanced GC tissues compared with early GC tissues (*P*<0.05, Table 1). A significant difference was also found between SETDB2 expression and pTNM stages (Stage I vs. II-IV, *P*<0.05). However, there were no significant correlations between SETDB2 expression and age, gender, histological classification or lymph node metastasis. Furthermore, SETDB2 expression was significantly associated with worse survival by Kaplan-Meier analysis (*P*<0.05, Figure 1C). Thus, SETDB2 overexpression might be potentially associated with clinical progression of GCs.

**Table 1:** Clinicopathological correlations of SETDB2 immunohistochemistry in 72 GC tissues

Clinicopathol group	No. of cases	SETDB2		*P*-value^1)^
		Positive n=30 (%)	Negative n=42 (%)	
*Age*				
≤ 60	28	12 (42.9)	16 (57.1)	0.870
> 60	44	18 (40.9)	26 (59.1)	
*Gender*				
Female	21	7 (33.3)	14 (66.7)	0.350
Male	51	23 (45.1)	28 (54.9)	
*Lauren's classification*				
Intestinal	36	17 (47.2)	19 (52.7)	0.339
Diffuse	36	13 (36.1)	23 (63.9)	
*Tumor invasion*				
EGC (pT1)	12	1 (8.3)	11 (91.7)	0.010*
AGC (pT2-4)	60	29 (48.3)	31 (51.7)	
*pT stage*				
pT1a	2	0 (0)	2 (100.0)	0.157
pT1b	10	1 (10.0)	9 (90.0)	
pT2	8	3 (37.5)	5 (62.5)	
pT3	24	13 (54.2)	11 (45.8)	
pT4a	25	11 (44.0)	14 (56.0)	
pT4b	3	2 (66.7)	1 (33.3)	
*Lymph node metastasis*				
Absent (N0)	30	10 (33.3)	20 (66.7)	0.187
Present	42	20 (47.6)	22 (52.4)	
*pTNM stage*				
I	17	3 (17.6)	14 (82.4)	0.260
II	23	11 (47.8)	12 (52.2)	
III	22	10 (45.5)	12 (54.5)	
IV	10	6 (60.0)	4(40.0)	
*pTNM stage*				
I	17	3 (17.6)	14 (82.4)	0.026*
II+III+IV	55	27 (49.1)	28 (50.9)	
I	17	3 (17.6)	14 (82.4)	0.044*
II+III	45	21 (46.7)	24 (53.3)	

1) *P*-values were determined by Pearson's chi-square test. The Spearman rank correlation analysis was performed for pT and pTMN stages.

* Statistically significant difference.

### Analyses of SETDB2 expression in GC cell lines

**Figure 2:**
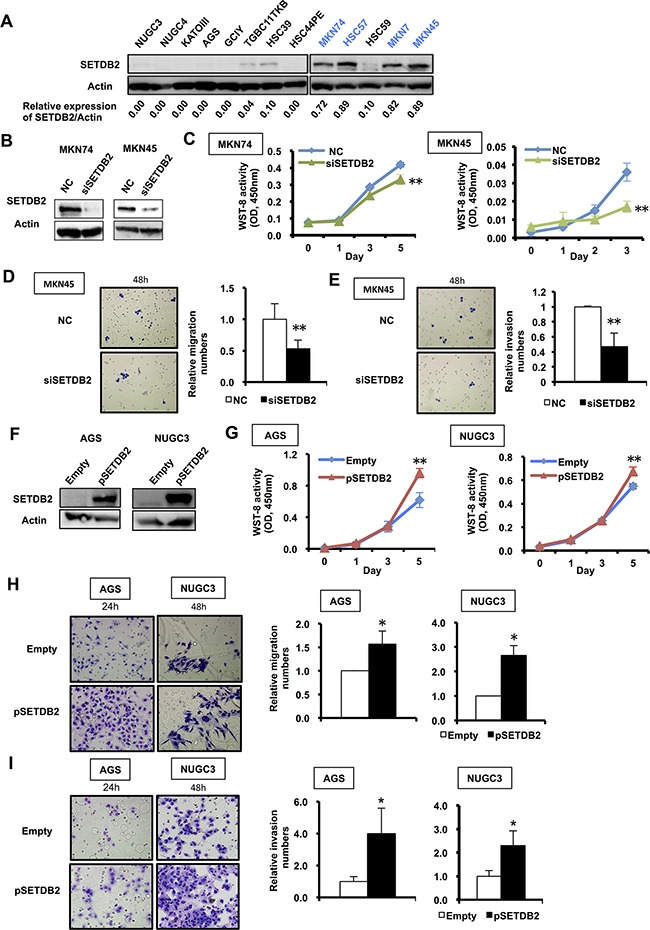
Functional analyses of SETDB2 in cultured GC cell lines **A.** Western blot analysis of the expression levels of SETDB2 protein in 13 GC cell lines. Actin expression was analyzed as an internal control. SETDB2 protein expression was quantified using Image J software, and then was normalized to Actin expression. GC cell lines were classified into SETDB2-high and -low expressing groups, which were determined by a cut-off value of 0.50. Accordingly, four GC cell lines highlighted in blue (high: HSC57, MKN7, MKN45 and MKN74) strongly expressed the SETDB2 protein, while the remaining nine cell lines exhibited no or weak SETDB2 expression (low). **B.** Western blot analysis of SETDB2 expression in GC cells with its knockdown. SETDB2 expression was suppressed in MKN74 and MKN45 cells by transfection with *SETDB2* siRNA (siSETDB2) compared with ones with negative control siRNA (NC). Actin expression was used as an internal control. **C.** Cell proliferation was quantified with a Cell Counting Kit-8 after transfection of *SETDB2* siRNA into MKN74 and MKN45 cells. ◆, (blue), negative control siRNA; ▲, (green), *SETDB2* siRNA transfection. Data are from 3 replicate experiments and error bars indicate standard deviation. Student's t-test; **: *P*<0.01. **D** and **E.** The cell migration and invasion were assayed using a control insert (D) and a Matrigel invasion chamber (E), respectively, in MKN45 cells transfected with siSETDB2 or control siRNA. Original magnification: x100. Student's t-test; **: *P*<0.01. **F.** Western blot analysis of SETDB2 overexpression in GC cells. AGS and NUGC3 were transfected with the empty vector (Empty) or the SETDB2 overexpressing vector (pSETDB2). Actin expression was analyzed as an internal control. **G.** Analysis of the cell proliferation of AGS and NUGC3 cells transfected with the SETDB2 overexpressing (▲, red), or the empty (◆, blue) vector. Data are from 3 replicate experiments and the error bars indicate standard deviation. Student's t-test; **: *P*<0.01. **H** and **I.** Analyses of cell migration and invasion abilities in AGS and NUGC3 with SETDB2 overexpression using a control insert (H) and a Matrigel invasion chamber (I). Original magnification: x100. Student's t-test; *: *P*<0.05.

We next examined SETDB2 protein expression levels in 13 GC cell lines using Western blot analysis. By densitometric quantification of the SETDB2 bands, we subsequently classified these cell lines into SETDB2 high- (HSC57, MKN7, MKN45 and MKN74) and low- (AGS, GCIY, TGBC11TKB, HSC39, HSC44PE, HSC59 NUGC3, NUGC4 and KATOIII) expression groups, which were determined by a cut-off value of 0.50 (Figure 2A). To evaluate the biological significance of SETDB2 expression, the effect of knockdown of *SETDB2* expressing using RNA-interference was analyzed. SETDB2 protein expression was efficiently suppressed by transfection of *SETDB2* siRNAs (siSETDB2) into MKN74 and MKN45 cells compared with its expression in negative control siRNA-transfected cells (Figure 2B). These two cell lines in which *SETDB2* was knocked down showed significant inhibition of cell proliferation compared with the cells transfected with the negative control siRNA (*P*<0.05, Figure 2C). Moreover, knockdown of *SETDB2* markedly decreased the cell migration and invasion rates of MKN45 cells (*P*<0.05, Figure 2D and 2E). In addition, we analyzed the effect of SETDB2 overexpression in AGS and NUGC3 cells by transfection with the SETDB2/pcDNA3 expression vector (Figure 2F). Cell growth, migration and invasion were enhanced in the GC cells with SETDB2 overexpression compared with the GC cells transfected with empty vector (*P*<0.05, Figure 2G-2I). Scratch assay also demonstrated that cell migration was significantly faster in AGS and NUGC3 cells with SETDB2 overexpression than those with empty vector transfection (*P*<0.05, Supplementary Figure S1A).

### The relationship between SETDB2 expression and global H3K9 methylation in GC cells

**Figure 3:**
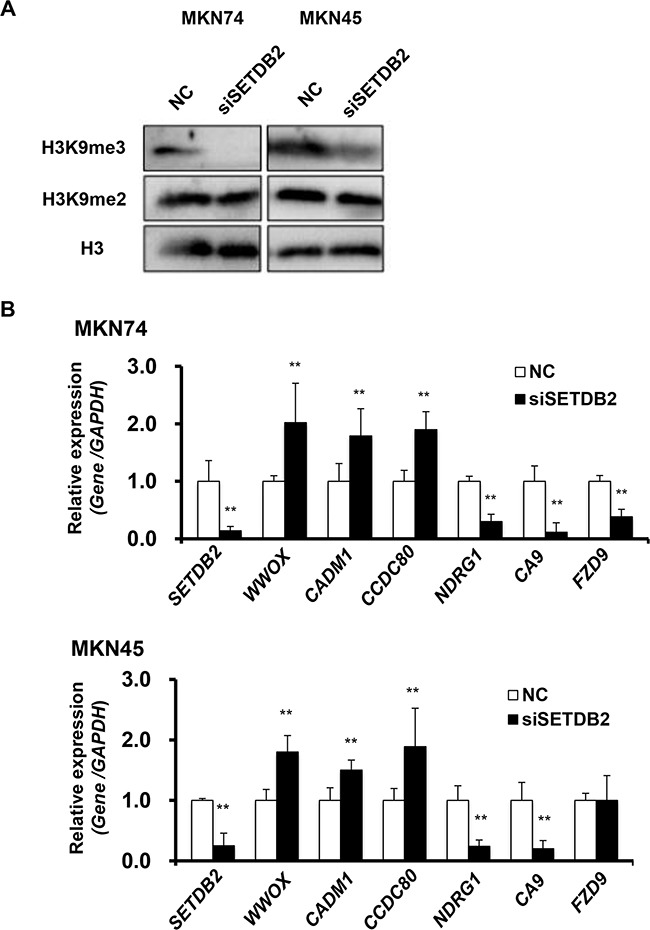
Analyses of the SETDB2 target genes in GC cells **A.** Western blot analysis of the global H3K9 methylation levels of MKN74 and MKN45 cells transfected with *SETDB2* (siSETDB2) or control (NC) siRNA. **B.** After knockdown of *SETDB2* in MKN74 cells, expressionaly altered six genes were selected as its downstream targets detected by microarray analysis. The mRNA expression levels of these genes were validated by real-time RT-PCR in MKN74 and MKN45 after knockdown of *SETDB2*. The white bars are cell lines transfected negative control siRNA and the black bars are cell lines transfected SETDB2 siRNA.

To investigate whether or not altered SETDB2 expression evokes H3K9 methylation change in GC cells, we compared global H3K9 methylation levels in MKN74 and MKN45 cells between *SETDB2* knockdown and control cells. Global H3K9me3 at the protein levels were decreased in both of these cell lines with knockdown of *SETDB2* by Western blotting, although global di- (H3K9me2, Figure 3A) and mono-methylation (H3K9me1, data not shown) levels were not changed. To further clarify whether decrease global H3K9me3 levels in our study were independently effects by SETDB2 knockdown or the secondary ones by other H3K9 tri-methyltransferase, we examined expression of SETDB1 and SUV39H1 in MKN74 and MKN45 cells with *SETDB2* knockdown. The mRNA expression of *SETDB1* and *SUV39H1* was not changed in MKN74 and MKN45 cells after SETDB2 knockdown (Supplementary Figure S2), suggesting that SETDB2 itself is correlated with global H3K9me3 levels in these two GC cell lines.

### Analyses of SETDB2 target genes in GC cells

To determine SETDB2 target genes, microarray analysis of MKN74 cells with *SETDB2* knockdown was performed. There were 520 genes that showed a greater than 1.5-fold change in expression level in MKN74 cells after *SETDB2* knockdown, of which 185 genes were up-regulated and 335 genes were down-regulated. Because *SETDB2* knockdown led to significant inhibition of cell growth, migration and invasion, we further selected six tumor- or differentiation-related genes (*WWOX, CADM1/TSLC1, CCDC80, NDRG1, CA9* and *FZD9*) that have been reported to show altered expression in several cancers including GCs [13–18]. Expression of *WWOX, CADM1/TSLC1* and *CCDC80* genes was up-regulated, while that of *NDRG1, CA9* and *FZD9* genes was down-regulated in MKN74 cells transfected with *SETDB2* siRNA compared with control cells by real-time and conventional RT-PCR analyses (Figure 3B and Supplementary Figure S3A). These results were consistent with the microarray data. *SETDB2* knockdown in MKN45 cells resulted in similar gene expression patterns except for expression of *FZD9* (Figure 3B and Supplementary Figure S3A).

### SETDB2 down-regulated the target genes through H3K9me3 in GCs

**Figure 4:**
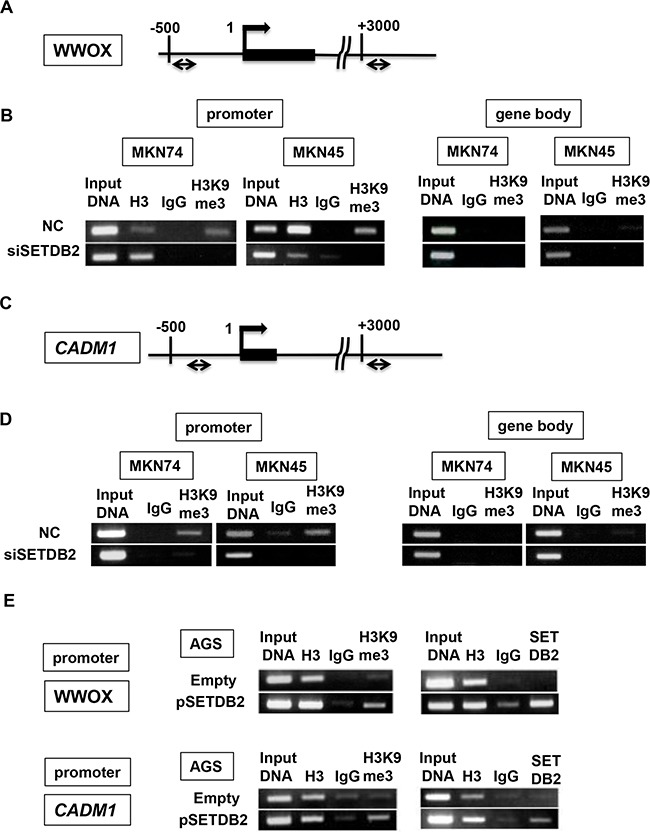
ChIP analysis of the promoter region of *WWOX* and *CADM1* expression in GC cells **A.** Schematic representation of the promoter region of the *WWOX* gene. ChIP assay was performed at the regions of the promoter (−493 to -325) and gene body (+3040 to +3206). **B.** ChIP assay was performed with anti-histone H3 and anti-H3K9me3 polyclonal antibodies, and normal rabbit IgG. The H3K9me3 levels were enriched at the *WWOX* promoter region (−493 to −325) in MKN74 and MKN45 cells with negative control transfection (NC) compared with ones with siSETDB2 transfection. The H3K9me3 was not detected at the gene body region (+3040 to +3206). Input DNA sample was used as an internal control. **C.** Schematic representation of the promoter region of the *CADM1* gene. **D.** ChIP assay was performed at the regions of the *CADM1* promoter (−359 to −207) and gene body (+3085 to +3289). E) H3K9me3 levels and SETDB2 binding at the promoter regions of *WWOX* and *CADM1* in AGS cells with SETDB2 overexpression.

SETDB2 had an H3K9me3 activity in our study (Figure 3A), and H3K9me3 is known to be associated with gene silencing [4]. We therefore focused on the three genes (*WWOX, CADM1 and CCDC80*) whose expression was elevated by *SETDB2* knockdown, and we examined how SETDB2 expression inhibited the expression of these genes in GC cells. ChIP assays showed that H3K9me3 levels at the promoter regions of *WWOX* and *CADM1* were greatly decreased in MKN74 and MKN45 cells with *SETDB2* siRNA transfection compared with negative control (Figures 4A-4D). In contrast, the H3K9me3 levels at a region approximately +3 kb downstream of the transcriptional start site (TSS) were not changed by *SETDB2* siRNA transfection compared with negative control transfection (Figures 4B and 4D, right panels). Conversely, AGS cells transfected with the SETDB2 expression vector showed increased H3K9me3 levels at the promoter region compared with cells transfected with the empty vector (Figure 4E, left panels). Furthermore, ChIP assays exhibited that SETDB2 was recruited to the promoter region of *WWOX* and *CADM1* in AGS cells after overexpression of SETDB2 (Figure 4E, right panels), suggesting that these two genes may be direct targets of SETDB2 in GC cells. Similar interaction of *CCDC80* with SETDB2, however, was not detected (data not shown).

**Figure 5:**
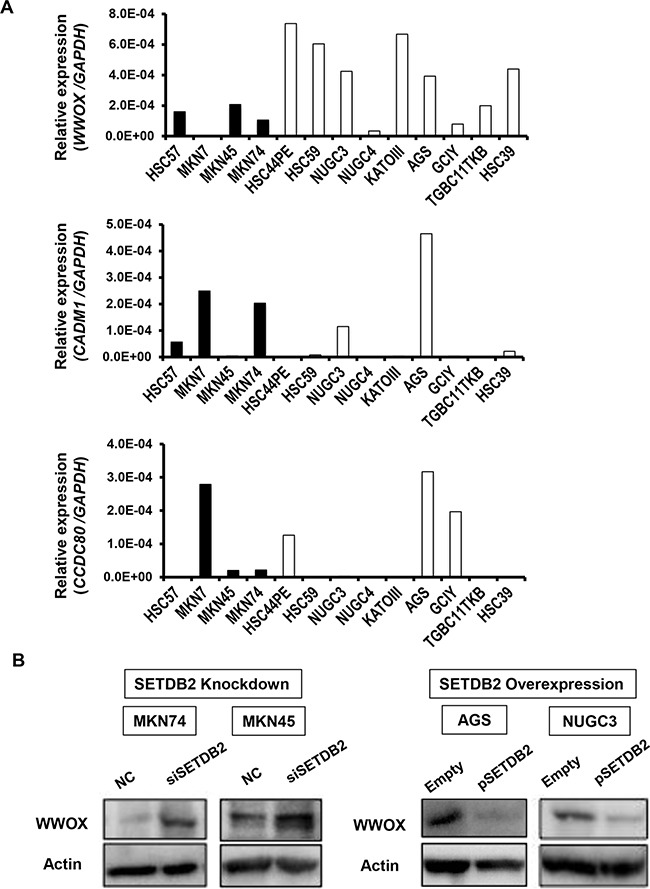
The relationship between SETDB2 and target gene expression in GC cell lines **A.** The expression levels of *WWOX*, *CADM1* and *CCDC80* mRNA were examined in 13 GC cell lines using real-time RT-PCR. The black bars are cell lines with high SETDB2 protein expression and the white bars are cell lines with low SETDB2 protein expression. **B.** WWOX protein expression levels were examined by Western blotting in GC cells with SETDB2 knockdown (left), and overexpression (right). Actin expression was analyzed as an internal control.

We next compared the expression levels of endogenous SETDB2 and its three target genes (*WWOX, CADM1* and *CCDC80*) in 13 GC cell lines using real-time and conventional RT-PCR analyses. The expression levels of *WWOX* in GC cell lines with high SETDB2 expression were lower than those with low SETDB2 expression, thereby displaying a tendency of *WWOX* expression to inversely correlate with SETDB2 expression (*P*=0.058), (Figure 5A and Supplementary Figure S3B). However, the expression levels of *CADM1* and *CCDC80* were not correlated with SETDB2 expression levels as assessed by either conventional RT-PCR (Figure 5A and Supplementary Figure S3B).

### Functional analysis of WWOX in GC cells

**Figure 6:**
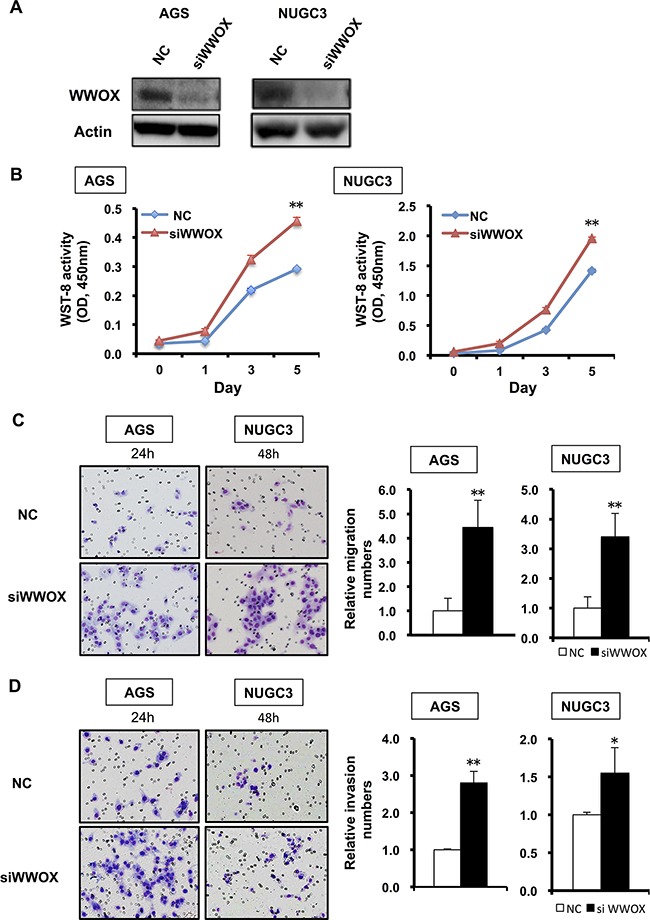
Functional analysis of WWOX in GC cells **A.** Western blot analysis of WWOX protein expression in AGS and NUGC3 cells transfected with negative control (NC) or *WWOX* siRNA (siWWOX). **B.** Cell proliferation was quantified with the Cell Counting Kit-8 after transfection of *WWOX* siRNA (▲, red) or negative control siRNA (◆, blue) into GC cells. Data are from 3 replicate experiments and error bars indicate standard deviation. Student's t-test; **: *P*<0.01. **C** and **D.** The cell migration (C) and the invasion (D) abilities of AGS and NUGC3 cells with *WWOX* siRNA were assayed using a control insert and a Matrigel invasion chamber, respectively. Original magnification: x100. Student's t-test; *: *P*<0.05, **: *P*<0.01.

Since *WWOX* is known to play a role as a TSG in various cancers [19, 20], we studied the relationship between SETDB2 and *WWOX*. *SETDB2* knockdown up-regulated WWOX protein expression in MKN74 and MKN45 cells, and, conversely, SETDB2 overexpression in AGS and NUGC3 cells repressed the protein expression of WWOX (Figure 5B). To assess whether or not the effects of SETDB2 overexpression in GC cells were mediated by reduced expression of WWOX, we performed knockdown of *WWOX* by transfection of its siRNA into AGS and NUGC3 GC cells (Figure 6A). The abilities of cell proliferation, migration and invasion were promoted after knockdown of *WWOX* in AGS and NUGC3 cells (*P*<0.05, Figure 6B-6D, Supplementary Figure S1B). It is noted that these effects were similar to those of SETDB2 overexpression in these cells (Figure 2G-2I, Supplementary Figure S1A).

## DISCUSSION

Previous studies have reported the expression changes of the HMT genes in various cancers [21–24]. The H3K9 tri-methyltransferase genes, *SETDB1* and *SUV39H1*, have been shown to be overexpressed in cancers, such as lung and liver cancers [25–27]. Overexpression of these two genes is related to cell proliferation and invasiveness, and Setdb1 accelerated melanoma formation in a transgenic zebrafish model, indicating that they have oncogenic properties [28]. In this study, we showed that the SETDB2 protein was highly expressed in GCs (41.7%), particularly in advanced GCs. Moreover, high SETDB2 expression in GCs was correlated with worse prognosis by Kaplan-Meier analysis (*P*<0.05). The *in vitro* studies using *SETDB2* knockdown and overexpressing cells of the present study demonstrated that SETDB2 promoted cell proliferation, migration and invasion of GC cells. Thus, SETDB2 expression and function detected in our study were similar to those of SETDB1 and SUV39H1 in cancers [5, 6], suggesting that SETDB2 may have oncogenic functions and overexpression of SETDB2 play roles in gastric cancer progression.

Although multiple transcriptional/translational regulatory mechanisms in SETDB1 and SUV39H1 have been identified [26, 27], the upstream mechanisms of SETDB2 are unknown. It has been reported that SETDB1 promoter region contains multiple SP1-binding sites and SETDB1 expression was enhanced by SP1, suggesting a significant relation between SETDB1 and TF bindings [26]. We observed that three putative myc-binding sites were but only one SP1 located at the SETDB2 promoter region by using UCSC Genome Bioinformatics Site (http://genome.ucsc.edu/cgi-bin/hgGateway). Knockdown of *c-myc* in MKN74 and MKN45 cells decreased SETDB2 at the mRNA and protein expression levels (Supplementary Figure S4A-S4C), indicating that c-myc may act as a transcriptional regulator of SETDB2 in these two GC cell lines. Besides, amplification of the *SETDB1* gene was found in lung and liver cancers [5, 25, 26], and SETDB1 and SUV39H1 were negatively regulated by miR-29 and miR-125b, respectively [26, 27]. Further studies are necessary to clarify the transcriptional and translational regulatory mechanisms of SETDB2 overexpression in GC cells.

The H3K9 methyltransferase genes mediated global and promoter/enhancer region of H3K9 tri- di- and mono- methylation [29–32]. Knockdown of *SETDB2* in MKN74 and MKN45 cells reduced global H3K9me3 (Figure 3A) but not H3K9me2 and H3K9me1 at the protein level, indicating that SETDB2 participates as an H3K9 tri-methyltransferase [10, 11]. High levels of global H3K9me3 protein have been shown in primary cancers including GCs [33, 34]. Therefore, we speculate that elevated H3K9me3 levels in GC tissues reported may be mediated in part by SETDB2 overexpression.

In addition, we observed that knockdown of *SETDB2* resulted in a decrease in H3K9me3 levels at the promoter regions of *WWOX* and *CADM1* genes, both of whose expression were increased by *SETDB2* knockdown. Ectopic SETDB2 in GC cells was recruited to the promoters of these two genes and increased H3K9me3 levels at the regions. Therefore, SETDB2 may repress target gene expression not only through increasing global H3K9me3 levels but also directly binding and inducing H3K9me3 activity on the promoter region of *WWOX* and *CADM1* in GC cells. In contrast, it is possible that *CCDC80* may be indirectly regulated by SETDB2, because SETDB2 did not bind to its promoter region.

Regarding the three genes (*WWOX, CADM1* and *CCDC80*) detected as SETDB2 target genes in our study, their loss or reduced expression has been reported in various cancers [35–38]. Here, loss or low expression of these three genes was found in GC cell lines examined (Figure 5A), and the expression levels of *WWOX* in GC cell lines showed a tendency to inversely correlate with SETDB2 expression. It is known that *WWOX* and *CADM1* but not *CCDC80* have dense CpG islands in their promoter regions and hypermethylation at the promoter regions of *WWOX* and *CADM1* has been reported in cancer cells [39, 40]. We examined the methylation status of *WWOX* and *CADM1* genes in 13 GC cell lines. No methylation of *WWOX* was found in any of the GC cell lines, while severe or partial methylation at the *CADM1* promoter was detected in six GC cell lines (data not shown). MKN74 and MKN45 cells exhibited no or partial *CADM1* methylation. Our data further support that *WWOX* and *CADM1* may be repressed by SETDB2 in GC cells.

In conclusion, our data indicated that SETDB2 is often over-expressed in GC tissues and cell lines. SETDB2 overexpression significantly accelerated cell proliferation, migration and invasion of GC cells. Moreover, SETDB2 restricted the expression of TSGs such as *WWOX*, through H3K9me3 at the their promoter regions. Since SETDB2 showed oncogenic functions in our study, it is possible that knockdown of *SETDB2* might be an effective therapeutic strategy for GCs.

## MATERIALS AND METHODS

### Cell lines and tissues samples

We studied 13 human GC cell lines (AGS, GCIY, HSC39, HSC44PE, HSC57, HSC59, KATOIII, MKN7, MKN45, MKN74, NUGC3, NUGC4 and TGBC11TKB). MKN7, MKN45, MKN74, GCIY and TGBC11TKB were purchased from RIKEN cell bunk, NUGC3 and NUGC4 were from Japanese Collection of Research Bioresources Cell Bank, and KATOIII and AGS were from ATCC (America Type Cell Collection). HSC39, HSC44PE, HSC57 and HSC59 were obtained from Dr. Kazuyoshi Yanagihara. All the cell lines were grown in RPMI 1640 medium (Wako, Saitama, Japan) or DMEM medium (Wako) supplemented with 10% fetal bovine serum and 1% penicillin/streptomycin at 37°C under 5% CO_2_ in a humidified incubator.

Cancer tissue samples were collected from 72 GC patients in an affiliated hospital of Tokyo Medical and Dental university. Informed consent was obtained from all of the patients, and the study was approved by the appropriate institutional review committee.

### RNA isolation, RT-PCR and real-time RT-PCR analysis

Total RNA was isolated using the Trizol reagent (Thermo Fisher Scientific Inc., Waltham, MA) or the RNeasy plus Minikit (QIAGEN, Valencia, CA). The isolated RNA (1 μg) was reverce-transcribed by using Super Script III Reverse transcriptase (Thermo Fisher Scientific Inc.). Glyceraldehyde-3-phosphate dehydrogenase (*GAPDH*) was amplified as an internal control. The primer sequences used are shown in Supplementary Table S1 (Supplementary Material).

### Western blot analysis

Total protein was extracted using RIPA buffer and was then electrophoresed on SDS-PAGE. Western blotting was conducted with mouse monoclonal anti-SETDB2 (1:500; M17, Abnova, Taipei, Taiwan), and anti-WWOX (1:300; sc-374449, Santa Cruz Biotechnology, Santa Cruz, CA): and rabbit polyclonal anti-H3 (1:4000; Active Motif, Carlsbad, CA), anti-H3K9me3 (1:2000; Active Motif) and anti-H3K9me2 (1:2000; Active Motif). Mouse monoclonal anti-Pan Actin (1:5000; MILLIPORE, Billerica, MA) was used as an internal control for Western blotting.

### Immunohistochemical staining

Immunohistochemistry (IHC) of formalin-fixed paraffin-embedded tissues was performed as described previously [41]. SETDB2 protein expression was evaluated in a set of 72 primary GC tissues by using rabbit polyclonal anti-SETDB2 (1:100; ab5517, abcam, Cambridge, UK). Sections in which > 10% of the cancer cells showed positive reactivity with SETDB2 were scored as positive. Similary, score of the SETDB2 positivity (>10% of cells) was used in non-cancerous stomach tissues.

### Plasmid construction

Full length SETDB2 was amplified from cDNA of MKN74 cells using RT-PCR. The primer sequences used were: 5′-CCGCTTAAGCTTAAGATGGGAGAAAAAAATGGCG-3′(sense) and 5′-CGAATTGATATCCACAAACAGGCGTTAGTTACAT-3′(antisense). After digestion with Hind III and EcoR V, the PCR product was subcloned into the pcDNA3 vector.

### Transfection

MKN74 and MKN45 cells with high SETDB2 expression were transfected with *SETDB2* siRNAs (final concentration, 50nmol; Hs_SETDB2_0144, Sigma-Aldrich Japan, Ishikari, Japan) or negative control siRNA (Mission_SIC-001, Sigma-Aldrich Japan) using electroporation (Neon^TM^ transfection System, Thermo Fisher Scientific Inc.). AGS and NUGC3 cells with low SETDB2 expression were transfected with the SETDB2/pcDNA3 expression vector (1 μg) or with the empty pcDNA3 vector using the FuGENE HD Transfection Reagent (Promega Corporation, Madison, WI). Transfection of *WWOX* siRNA (Hs_WWOX_9365, Sigma-Aldrich Japan) was conducted in GC cells by electroporation. Transfected cells were harvested after 48 or 72 hours of culture, and total RNA and protein was then extracted.

### Cell growth assay

The effects of *SETDB2* knockdown and overexpression on cell proliferation were quantified using the Cell Counting Kit-8 (Dojindo, Kumamoto, Japan). MKN74 (4×10^3^ cells/well) and MKN45 (3×10^3^ cells/well) cells were transfected with *SETDB2* siRNA or negative control siRNA. AGS (1×10^3^ cells/well), and NUGC3 (1×10^3^ cells/well) cells were transfected with the SETDB2/pcDNA3 or empty pcDNA3 expression vector. Moreover, the cell proliferation rates of AGS and NUGC3 cells transfected with *WWOX* siRNA were examined. GC cells transfected with siRNA or empty vector were seeded onto 96-well plates, and their cell proliferation was then determined by WST-8 activity.

### Transwell migration and invasion assays

MKN45, AGS and NUGC3 cells were transfected with *SETDB2* siRNA, *WWOX* siRNA, full length SETDB2/pcDNA3 or the empty/pcDNA3expression vector. After 48 hours, MKN45 (4×10^3^ cells/0.5ml), AGS (3×10^3^ cells/0.5ml) or NUGC3 (5×10^3^ cells/0.5ml) cells were seeded in a control insert or a Matrigel invasion chamber (BD Biosciences, Franklin Lakes, NJ) [9]. Medium containing 10% FBS in the lower chamber served as chemoattractant. After 48 hours, the non-invaded cells were gently removed with a cotton swab and the migrated and invaded cells located on the lower side of the chamber were stained with crystal violet.

### Scratch wound healing assay

At 48 hours after transfection of AGS and NUGC3 cells with the SETDB2 expression vector or with *WWOX* siRNA, the cells were seeded in 6 well plates at a density of 4×10^5^ cells/well and cultured to confluence. A scratch was made through the cell monolayer using a pipet tip. To monitor the migration of cells into the wounded area, photographs of the wounded area were taken immediately after the scratch was made (0 hour) and after then 18 hours later. The relative wound healing range was determined [9].

### Microarray analysis

MKN74 cells were transfected with *SETDB2* siRNA or negative control siRNA, and were then grown for 48 hours. Total RNA was extracted using RNEasy and cDNA microarray analysis was conducted by DNA Chip Research Inc. (Kanagawa, Japan) using the Agilent Human GE 4×44K v2 Microarray (Platform ID: GPL13497). The raw data have been deposited in NCBI Gene Expression Omnibus (GEO) under the accession number GSE76135.

### Chromatin immunoprecipitation (ChIP) assay

MKN74 and MKN45 cells were transfected with *SETDB2* siRNA or negative control siRNA for 48 hours. ChIP assays were performed using the ChIP-IT Express Kit (Active Motif) according to the manufacturer's instructions [9]. AGS cells were transfected with the SETDB2/pcDNA3 or empty/pcDNA3 expression vector. Antibodies used were anti-H3 (Active Motif), anti-H3K9me3 (Active Motif), Normal Rabbit IgG (No. 2729, Cell Signaling Technology, Danvers, MA) and anti-SETDB2 (No. 5517, abcam).

### Statistical analyses

The Mann-Whitney's U-test, c^2^ and Fisher's exact tests were used to compare the values for the test and control samples. Each transfection experiment was repeated at least in triplicate. Patient survival rate was calculated by the Kaplan-Meier method. A value of *P*<0.05 was determined as statistically significant.

## SUPPLEMENTARY MATERIAL FIGURES AND TABLE





## Abbreviations

ChIP: Chromatin immunoprecipitation
GC: gastric cancer
HMT: histone methyltransferase
H3K9me3: histone H3 lysine 9 tri-methylation
IHC: immunohistochemistry
RT-PCR: reverse transcription-polymerase chain reaction
TSGs: tumor suppressor genes
TSS: transcriptional start site.
