# Supplementary material for: Oncogenic roles of the SETDB2 histone methyltransferase in gastric cancer
Source: Oncotarget. 2016 Aug 26;7(41):67251–65. doi: 10.18632/oncotarget.11625 (PMC5341872; doi:10.18632/oncotarget.11625)
Supplement: Supplementary file 1 [file oncotarget-07-67251-s001.pdf]

# Oncogenic roles of the SETDB2 histone methyltransferase in gastric cancer

## SUPPLEMENTARY FIGURES AND TABLE

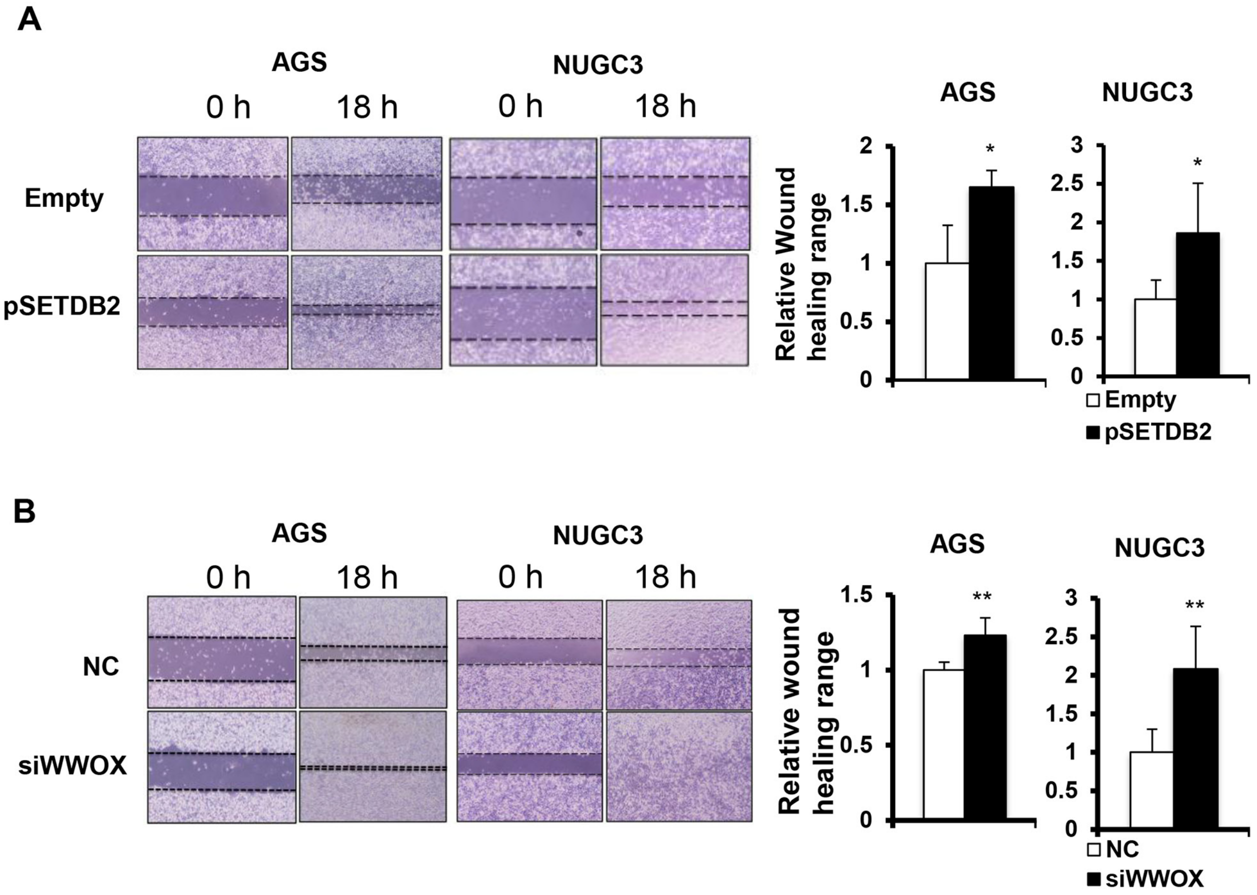


**Supplementary Figure S1: Wound healing assay of cell migration of AGS and NUGC3 cells. A** and **B.** The cell migration of AGS and NUGC3 cells with SETDB2 overexpression, A) or with *WWOX* siRNA, B), was assessed using a wound healing assay. Student's t-test; *: *P*<0.05, **:*P*<0.01.

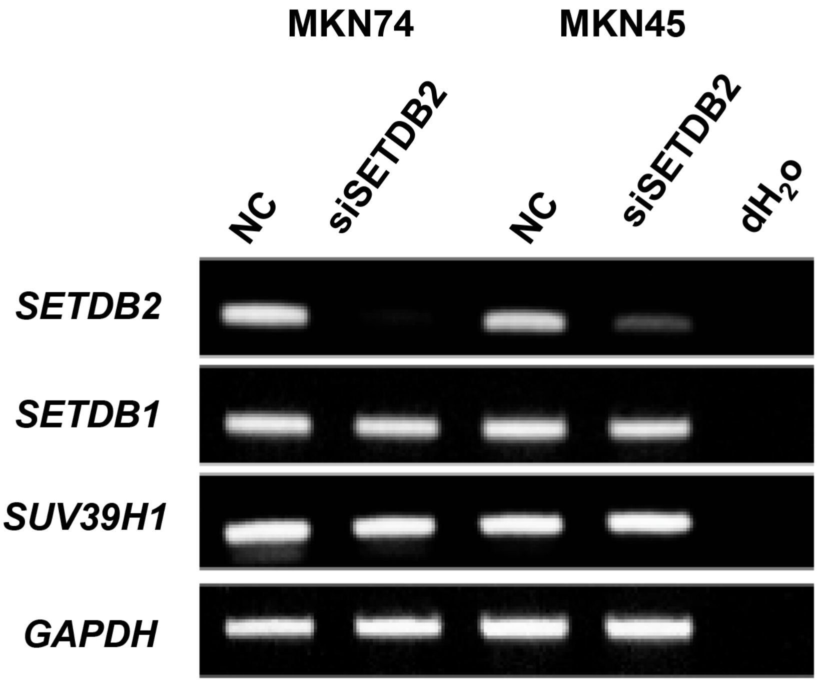


**Supplementary Figure S2: Expression of H3K9 tri-methyltransferase genes in GC cells with *SETDB2* knockdown.** The mRNA expression levels of two H3K9 tri-methyltransferase genes, *SETDB1* and *SUV39H1,* were examined by RT-PCR in MKN74 and MKN45cells after knockdown of *SETDB2*. d$H_2O$ was used as a negative control of RT-PCR.

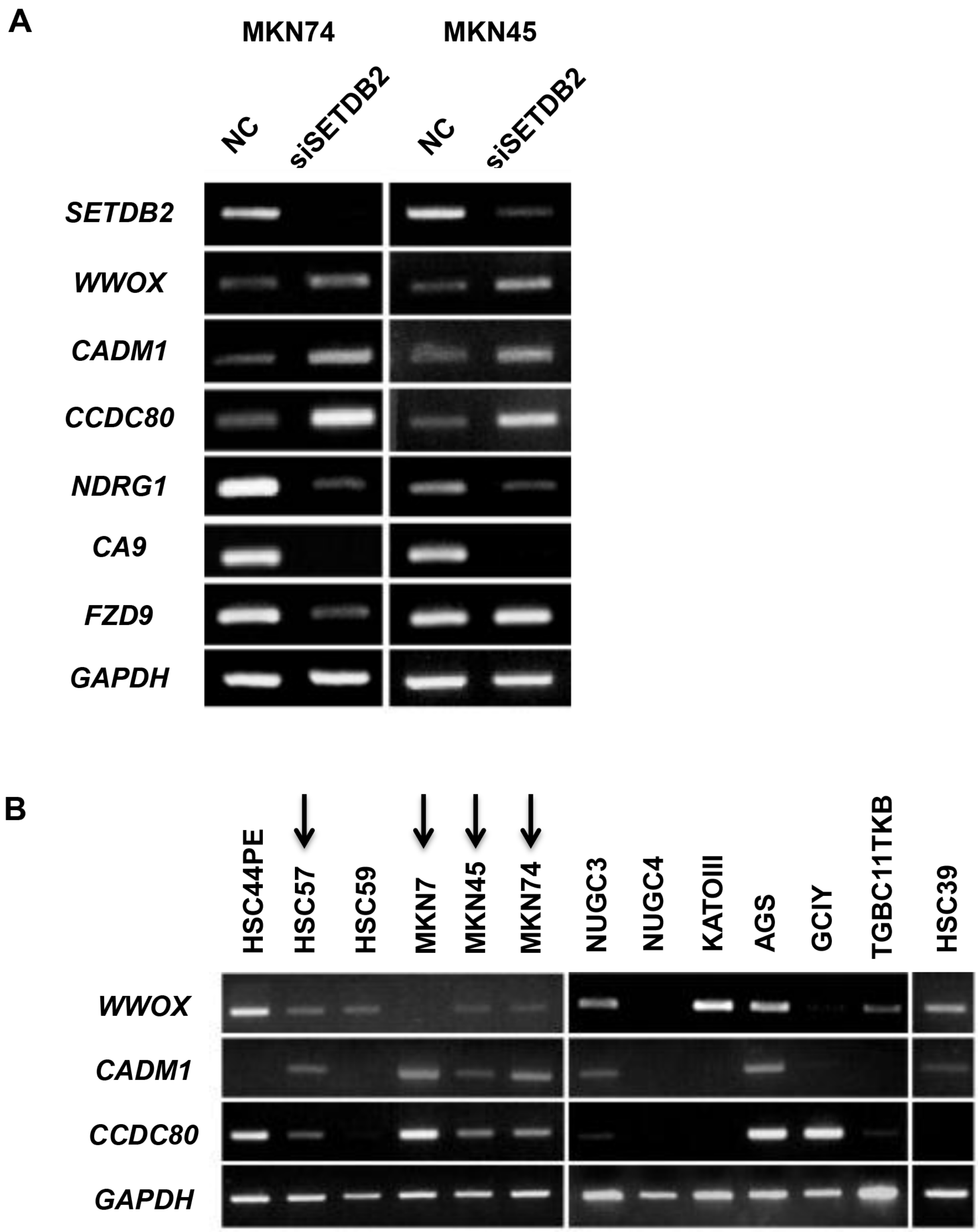


**Supplementary Figure S3: Analyses of the SETDB2 target genes in GC cells. A.** After knockdown of *SETDB2* in MKN74 cells, expressionaly altered six genes were selected as its down stream targets detected by microarray analysis. The mRNA expression levels of these genes were validated by conventional RT-PCR in MKN74 and MKN45 after knockdown of *SETDB2*. **B.** The mRNA expression levels of the tumor suppressive genes, *WWOX, CADM1,* and *CCDC80*, were examined in 13 GC cell lines using RT-PCR. The GC cell lines indicated by arrows are high SETDB2 expression cells, as shown in Figure 2A.

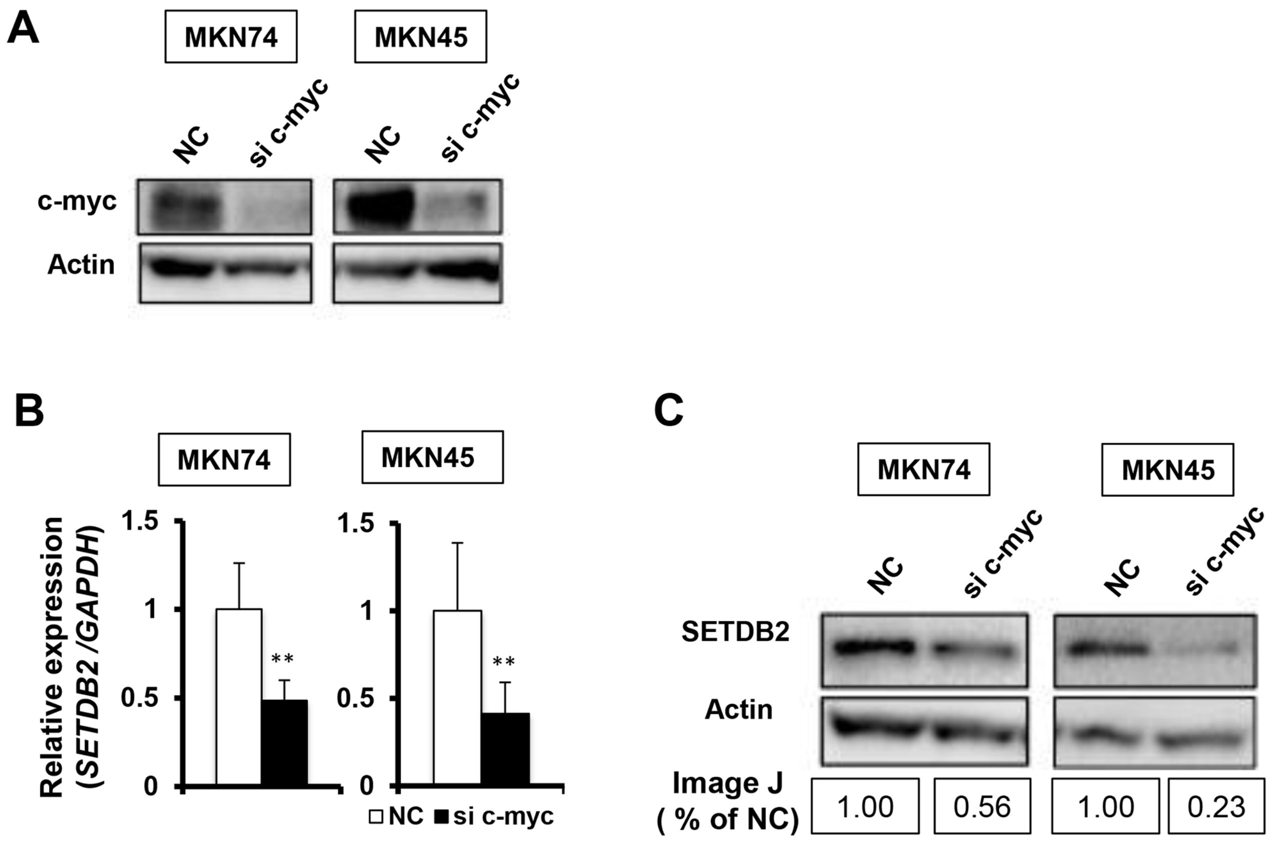


**Supplementary Figure S4: Expression of SETDB2 in GC cells with *c-myc* knockdown. A.** Western blot analysis of c-myc protein expression in MKN74 and MKN45 cells transfected with negative control (NC) or *c-myc* siRNA (si c-myc, SASI_Hs01_00222676, Sigma-Aldrich Japan). **B** and **C.** The SETDB2 expression levels were examined by real-time RT-PCR (B) and by Western blotting (C) in GC cells with c-myc knockdown. *GAPDH* and Actin expression was analyzed as an internal control.

**Supplementary Table S1: Representative primer sequences in this study[1)].**

**See Supplementary File 1**
