# Supplementary material for: Oncogenic roles of the SETDB2 histone methyltransferase in gastric cancer
Source: Oncotarget. 2016 Aug 26;7(41):67251–65. doi: 10.18632/oncotarget.11625 (PMC5341872; doi:10.18632/oncotarget.11625)
Supplement: Supplementary file 2 [file oncotarget-07-67251-s002.docx]

Table S1. Representative primer sequences in this study1) 

| Type of  PCR | Genes GenBank accession No. | Strand | Seaquences | Size of the PCR  products (bp) |
| --- | --- | --- | --- | --- |
| qRT-PCR | SETDB2 NM_031915 | forward reverse | GCCCAAATCTCTTGGTACAGAATG GATTTCCTTCTCAGGCACAGTCC | 152 |
| WWOX NM_16373 | forward reverse | TTCTACCTTGTCCAGCTCCTCC GTCCAGTTTTCCCAAGGAGTCG | 120 |
| CADM1 NM_014333 | forward reverse | CAGATAATGGTACATACCGCTG GTTGTGGGAGGAGGGATAGTTG | 109 |
| CCDC80 NM_199511 | forward reverse | GCCACATAACCATTCTGAAGC GTCTTCTCGCTCAACAACAGAG | 101 |
| NDRG1 NM_006096 | forward reverse | GCTGAGGCCTTCAAGTACTTCG GTGCCATCCAGAGAAGTGACG | 116 |
| CA9 NM_001216 | forward reverse | CCAGTCCAGCTGAATTCCTG CTTCTGTGCTGCCTTCTCATC | 143 |
| FZD9 NM_003508 | forward reverse | CAACACAGAGAAGCTGGAGAAG CAGAAGTCCATGTTGAGGCG | 120 |
| GAPDH NM_001256799 | forward reverse | TGCACCACCAACTGCTTAGC GGCATGGACTGTGGTCATGAG | 87 |
| ChIP | WWOX promoter NM_16373 | forward reverse | GTGCTCCGTAAACATCAGTC CCTGATTTCCTGGTTTTAGAG | 169 |
| WWOX Body NM_16373 | forward reverse | GGCTCTGGAGAATTAAAGGGAC GCTTACATGTCACTGCCCAAG | 167 |
| CADM1 promoter NM_014333 | forward reverse | GTGAGTGACGGAAATTTGCAAC GGTCTAGCTTCTTGTACACC | 153 |
| CADM1 Body NM_014333 | forward reverse | GGATTAGAGAAGATGGCTGGTG GATGCAGGGAAAGGCAACAATAAC | 154 |
| RT-PCR | SETDB2 NM_031915 | forward reverse | CTACAGCATGTCAAAGACAGCAGG TCTTCTCAGGCACAGTCCCAGC | 310 |
| SETDB1 NM_001145415 | forward reverse | AGAACACACGCCAATTCTATGATGG CAGCAACAGAGTAGCTCCTTGCC | 258 |
|  | SUV39H1 NM_001282166 | forward reverse | CATCTCCCACTTTGTCAACCACAG ATACGGACCCGCTTCTTAGGGGA | 243 |
| WWOX NM_16373 | forward reverse | CGCAGTGCATCCTGGAAATATG GTCTCTTCGCTCTGAGCTTCTG | 221 |
| CADM1 NM_014333 | forward reverse | GTGGAAGGTGAGGAGATTGAAG GAGGATAAGTCATCTGAATGTGC | 289 |
| CCDC80 NM_199511 | forward reverse | GCCACATAACCATTCTGAAGC CGTACACAATCACCATGGACC | 240 |
| NDRG1 NM_006096 | forward reverse | GCTGAGGCCTTCAAGTACTTCG GAGTTGGGGGTGATGTCCAG | 234 |
| CA9 NM_001216 | forward reverse | CTGGTGATCTCCGGCTACAG CTTCTGTGCTGCCTTCTCATC | 253 |
| FZD9 NM_003508 | forward reverse | GCTCAAAATTTTCATGTCACTGGTG GGTCCGTCTTAGTCATGTGCAAG | 215 |
| GAPDH NM_001256799 | forward reverse | GACCACAGTCCATGCCATCAC GTCCACCACCCTGTTGCTGTA | 454 |

1) The primer sequences of the genes used by other PCR in this study and their conditions are available upon request.
